# Predicting homeless people’s perceived health after entering the social relief system in The Netherlands

**DOI:** 10.1007/s00038-017-1026-x

**Published:** 2017-08-18

**Authors:** Jorien van der Laan, Barbara van Straaten, Sandra N. Boersma, Gerda Rodenburg, Dike van de Mheen, Judith R. L. M. Wolf

**Affiliations:** 1 Department of Primary and Community Care, Radboud University Medical Center, Impuls - Netherlands Center for Social Care Research, Nijmegen, The Netherlands; 2 Amsterdam University of Applied Sciences, Amsterdam, The Netherlands; 3 Erasmus Medical Centre, Rotterdam, The Netherlands; 4 IVO Addiction Research Institute, Rotterdam, The Netherlands; 5 Tilburg University, Scientific Centre for Care and Welfare (Tranzo), Tilburg, The Netherlands

**Keywords:** Cohort studies, Homelessness, Perceived health, Competence, Psychological distress

## Abstract

**Objectives:**

We explored whether changes in the perceived health of homeless people after entering the social relief system (SRS) in The Netherlands were predicted by housing, income, hours of work, social support, unmet care needs, arrests, physical and mental health, substance use, and experiences of autonomy, competence and relatedness, in addition to perceived health at baseline, demographics, suspected intellectual disability, the duration of homelessness and the company of children in the shelter facility.

**Methods:**

A hierarchical regression analysis was used to explore the significant predictors of the perceived health of 344 homeless persons 18 months after entering the social relief system.

**Results:**

A decrease in psychological distress and an increase in hours of (paid/voluntary) work as well as competence predicted a better perceived health.

**Conclusions:**

Perceived health is not only influenced by objective circumstances related to work and mental health, but also self-determination, as shown by the influence of competence. Services should aim to reduce psychological distress of homeless people, support them in increasing their working hours and focus on strengthening their competence.

## Introduction

Homeless people experience more difficulties than the general population in meeting even the most basic needs in life, which is reflected in their having a lower perceived health (Gadermann et al. 2014). There is mounting evidence that housing (Hwang and Burns 2014; Kertesz et al. 2005), income, work (Daiski 2007) and social support (Hwang et al. 2009) are all associated with better health, while mental and physical health complaints and being arrested by the police are associated with poorer perceived overall health (Gadermann et al. 2014). Furthermore, findings show that the health of homeless people can be improved through changes in their living conditions (Hwang et al. 2011; Kertesz et al. 2005) and by offering service programs (Gilmer et al. 2010; Schanzer et al. 2007). Promising programmes seem to owe their success to the strengthening of autonomy (O’Campo et al. 2009). Factors, such as suspected intellectual disability (ID) (Mercier and Picard 2011), a history of homelessness (Kertesz et al. 2005), and being accompanied by children (Grant et al. 2013) also influence health in homeless people, but these factors are not modifiable. Therefore, for the development of effective service programs it is not only important to understand whether perceived health can be improved by potential modifiable factors such as income, but also to be aware of the relative influence of each of these factors on perceived health. Such insights will be useful in prioritizing the types of support which can be offered.

To date, no studies have looked at modifiable factors and their influence on perceived health over time. This longitudinal study, therefore, aims to: (1) explore changes in the perceived health of the homeless population over a period of 18 months after entering the social relief system (SRS) in the four major cities of The Netherlands; (2) examine whether these changes are predicted by changes in the following modifiable variables: housing, income, hours of (paid/voluntary) work, social support, unmet care needs, arrests, physical and mental health, substance use, and experiences of autonomy, competence and relatedness, in addition to perceived health at baseline, demographics, suspected ID, the duration of homelessness and the company of children in the shelter facility.

## Methods

### Design and participants

This study is part of a longitudinal multi-site cohort study following homeless persons for a period of 30 months, starting from the moment they reported to a central access point for social relief in 2011 in one of the four major cities of The Netherlands (Amsterdam, The Hague, Rotterdam and Utrecht). All homeless persons are required to follow this procedure to gain access to the facilities of the SRS, including health care and accommodation. At baseline, all 513 study participants satisfied the criteria for entering the SRS, including being aged ≥18, having abandoned their home situation, and being unable to hold their own in society. The participants, consisting of both homeless young adults (aged 18–22) and adults (aged 23 or older) were divided over the four cities in accordance with the inflow of homeless people at the central access points. The participants in this study were compared with the total group of homeless adults and young adults who registered at one of the central access points in the four major cities in 2011. Adult participants were representative in terms of age and gender. Young adults were representative in terms of age but males were overrepresented (60.2% younger males in the cohort vs. 49.2% younger males in the total group).

Of the initial cohort of 513 participants, 344 (67.1%) were also interviewed for the two follow-up measurements. We compared respondents (*n* = 344) with non-respondents (*n* = 169) on demographic variables (age, gender, education, ethnicity) at baseline. Compared to respondents, non-respondents were on average younger (32.7 vs. 37.9 years) and more often had a non-native Dutch background. No selective response was found with respect to gender and education. For the purposes of this study, we excluded 15 participants who were accepted at the SRS because of a forthcoming eviction but were still actually housed. One participant who did not report his or her housing situation at baseline was also excluded.

The majority of the remaining 328 participants were male (74.1%) and their mean age was 37.96 years (SD = 13.08). The majority (59.6%) was non-native Dutch, of which only six participants were unable to complete the interview in Dutch and were, therefore, interviewed in English (*n* = 2), Spanish (*n* = 2) or Arabic (*n* = 2). Most participants had a low educational level (77.3%). A suspected ID was found in 30.6% of the participants. When entering the SRS, the mean life time duration of homelessness was 31.54 months (SD = 45.05, median = 12 months) and 7.6% of the participants were accompanied by one or more children.

### Procedure

At the start of the study in January 2011, potential participants were approached either at a central access point for social relief or at their temporary accommodation. When a potential participant agreed to take part a trained interviewer scheduled an appointment at the participant’s location of choice. All the participants gave their written informed consent. They were interviewed face-to-face using a structured questionnaire (mean duration 90 min) and received €15 for their participation. Participants were later contacted for two follow-up interviews six and 18 months after the baseline interview using the contact information provided at baseline. The follow-up interviews were similar to the baseline interview and participants received €20 and €25, respectively for these two sessions. All the data in this paper were derived from two sources, namely the baseline and 18 months interviews, with the exception of the suspected ID details, which came from the 6 month interview.

### Measures

#### Demographic characteristics

Age, gender, ethnicity (native or non-native Dutch) and educational level (varying from lower secondary to higher education).

#### Non-modifiable predictor variables

Lifetime homelessness was measured in the total number of months. We assessed whether participants were accompanied by their children in the shelter facility when entering the SRS. Suspected ID was assessed by a brief screening index, the Dutch version of the Hayes Ability Screening Index (HASI) (Hayes 2000), that gives an indication of whether a person has an ID (IQ < 70). The HASI shows a significant correlation with other psychometric tests measuring cognitive ability (0.63 for the Kaufman Brief Intelligence Test and 0.50 for the Vineland Adaptive Behavior Scales (Hayes 2000).

#### Modifiable predictor variables

The Dutch abbreviated version of the Lehman QoL Interview (Lehman et al. 1995; Wolf et al. 2002) was used to measure the following variables:

- housing status by asking participants whether they were living in their own apartment or house or were living with family, friends or acquaintances on a permanent basis, staying in an emergency shelter or night shelter; in transitional accommodation; living rough; temporarily staying with friends, relatives or acquaintances; residential care or supported accommodation (long stay); staying in a medical institution, drug habilitation institution or psychiatric hospital; staying in a correctional or penal institution; living in residential care or supported accommodation for people with mental health or substance abuse problems at the time of the interview. Housing status was marked as independent when participants were living in their own apartment or house or were living with family, friends or acquaintances on a permanent basis (yes = 1 or no = 0);
- income by assessing the adequacy of finances to cover costs for food, clothing, housing, traveling around the city and social activities (yes = 1 or no = 0), respectively, during the last month. The mean number of covered expenditures (ranging from 0 to 5) was calculated;
- hours of (paid/voluntary) work per week and (for descriptive purposes) degree of satisfaction with work and daily activities at the time of the interview;
- the number of arrests in the past year.

The psychometric properties of the brief version of the Lehman’s QoL Interview are good and comparable to the full interview version (Lehman et al. 1995). Moreover, the interview has been successfully applied in research in homeless populations (Krabbenborg et al. 2016; Wolf et al. 2001).

The availability of social support was assessed using items derived from the Medical Outcome Study (MOS) Social Support Survey (Sherbourne and Stewart 1991). Participants indicated how frequently different kinds of social support were available to them through family and friends or other acquaintances, on a scale ranging from *None of the time* (1) to *All of the time* (5). A family measure and a friends and acquaintances measure were constructed by averaging across items. The MOS Social Support Survey showed high convergent and discriminant validity and internal consistency. At baseline and follow-up, respectively, Cronbach’s *α* were .91. and .95 for family and .89 and .94 for friends and acquaintances.

Unmet care needs were assessed using a questionnaire developed by Impuls—Netherlands Center for Social Care Research, with response categories based on the Short Form Quality of Life and Care questionnaire (Wennink and van Wijngaarden 2004). Care needs were investigated using six life domains: living situation, finances, daily activities, physical health, mental health and social contacts. Two questions were asked in each domain: “Do you want help with …?” and “Do you get help with …?” at the time of the interview. An unmet care need variable was then created for each domain which scored affirmatively when participants indicated needing help but not receiving any. All unmet care needs were added up to achieve a total unmet needs variable, ranging from 0 to 6. The questionnaire was used successfully in research among homeless young adults (Krabbenborg et al. 2016).

To measure physical complaints, self-reported physical complaints during the previous 30 days were assessed using a list of twenty categories of complaints. This included fourteen categories based on the International Classification of Diseases (ICD) (World Health Organisation 1994) and five categories of common complaints in homeless people (visual, auditory, dental problems, foot problems, fractures) (Levy and O’Connell 2004) and a final category ‘health-related complaints not previously mentioned’. A ‘physical complaints’ measure was created by adding up the number of categories in which complaints were reported (range 0–20).

The number of days of alcohol use (at least five units per day) and cannabis use during the previous month were assessed using a module from the European version of the Addiction Severity Index (Europ-ASI, version III (Kokkevi and Hartgers 1995)). The ASI shows satisfactory results for their reliability and validity in studies among substance-abusing populations (Kokkevi and Hartgers 1995). The alcohol use and cannabis use variables could range from 0 to 31 days. No other substances were taken into account due to the low prevalence rates (<5%) in this population (Van Straaten et al. 2016a).

Psychological distress was measured on a scale ranging from *Not at all* (1) to *Extremely* (5), using the Dutch translation of the Brief Symptom Inventory 18 (BSI-18) (Derogatis 2001). A total psychological distress score was calculated by averaging across all 18 items. Cronbach’s *α* were .92 and .91 for the baseline and follow-up measurement. The BSI is a frequently-used measure to evaluate psychological distress in studies among homeless populations (Ball et al. 2005; Krabbenborg et al. 2016) and the Dutch version of the BSI demonstrates good reliability and validity (De Beurs and Zitman 2005).

Experiences of autonomy, competence and relatedness were measured by a Basic Psychological Needs questionnaire (Johnston and Finney 2010). The questionnaire consists of three subscales and participants were asked to indicate their agreement with 21 items on a seven-point Likert scale, ranging from *Not true at all* (1) to *Definitely true* (7). Each subscale reflects the extent to which a particular need is satisfied. At baseline and follow-up, respectively, Cronbach’s *α* for autonomy was .65 and .69, for competence .61 and .66 and for relatedness .72 and .72. This instrument has been used successfully in a study among homeless young adults (Krabbenborg et al. 2016).

#### Perceived health

The Dutch abbreviated version of the Lehman’s Quality of Life Interview (Lehman et al. 1995; Wolf et al. 2002) was used to measure perceived health. Three items were employed to assess perceived health: “How do you feel about: your health in general?/your physical condition?/your emotional well-being?”. Results were measured on a scale ranging from *Terrible* (1) to *Delighted* (7), and the scores were constructed by averaging the scores of the three items. At baseline and follow-up perceived health (Chronbach’s *α* = .77 and *α* = .73) showed sufficient reliability.

### Data analysis

To create the modifiable predictor variables, the scores at baseline were subtracted from the scores at the 18 months follow-up. Changes in the variables were explored by paired *t* tests. An univariate and a multivariate regression analysis were performed to examine predictors of perceived health at 18 months follow-up. Modifiable variables that were significantly univariately related to perceived health were selected for the hierarchical regression analysis. Baseline scores of perceived health were entered in the first step followed by the demographic variables (step 2), the non-modifiable variables (step 3) and modifiable variables (step 4). By doing this we were able to establish whether the modifiable variables accounted for a significant amount of incremental variance in the outcome variable. As the presence of missing values on either one of the variables would substantially reduce the final number of cases included in the analyses, missing values on individual variables of a scale were substituted with the mean score of the particular scale of the participant (when no more than 30% of the items were missing (Shrive et al. 2006)). All cases with missing values were deleted for the analysis of the univariate relationships. Missing data on the variables in the hierarchical regression analysis were imputed using the mean value of respondents without a missing value to prevent a possible bias that could occur if we had only selected those participants who chose to answer all the questions. Therefore, correlations involving the variable(s) that are imputed are attenuated, giving a conservative estimation of the relations in the analysis. All statistical analyses were conducted with using the IBM SPSS Statistics 20 programme.

## Results

### Changes from entering the SRS to 18 months later

There were improvements in the following areas between baseline and the 18 months follow-up: perceived health, income, hours of (paid/voluntary) work, social support by family and by friends and acquaintances, as well as experiences of autonomy, competence, and relatedness (Table 1). At baseline, all participants were homeless, while 18 months later 43.7% were independently housed. Other participants were housed in residential facilities for the homeless (29.8%), temporary accommodation (10.8%), with friends, family or acquaintances (short term) (8.3%), in institutions (6.2%) or were living on the streets (1.2%). One and a half years after entering the SRS, 44.6% (*n* = 146) reported working one hour or more per week. Participants were often in paid employment with a labour agreement (15.2%) or doing voluntary work (21.6%). Participants reported being *Mostly satisfied* to *Pleased* with their work (*M* = 5.85, SD = 1.21) and the workplace (*M* = 5.90, SD = 1.08).

**Table 1:** Perceived health, housing, income, hours of (paid/voluntary) work, social support and unmet care needs at baseline and 18 months follow-up, The Netherlands 2011–2013

Variables	At baseline	At 18 months follow-up	Change	Paired *t* test/McNemar test	Cohen’s *d*
		*M* (SD) or *n* (%)		*T* (*df*) or *Χ* ^2^	
Perceived health (*n* = 325)	4.70 (1.46)	5.07 (1.29)	0.37 (1.37)	4.89 (324)***	0.27
Independently housed (% yes) (*n* = 328)	0 (0.0%)	142 (43.3%)	142 (43.7%)	n.a.	
Income (*n* = 325)	2.16 (1.78)	3.01 (1.57)	0.85 (2.08)	7.37 (324)***	0.41
Hours of work (*n* = 317)	6.27 (11.52)	10.74 (14.86)	4.47 (17.00)	4.69 (316)***	0.26
Social support family (*n* = 313)	2.84 (1.32)	3.42 (1.45)	0.58 (1.37)	7.41 (312)***	0.42
Social support friends (*n* = 328)	3.11 (1.10)	3.46 (1.19)	0.34 (1.24)	5.02 (327)***	0.28
Unmet care needs (*n* = 327)	1.34 (1.27)	0.66 (1.02)	−0.68 (1.44)	−8.58 (326)***	−0.47
Arrests (*n* = 318)	0.79 (2.62)	0.25 (0.70)	−0.54 (2.49)	−3.89 (317)***	−0.22
Physical health complaint (*n* = 328)	3.01 (2.40)	1.90 (2.05)	−1.11 (2.20)	−9.16 (327)***	−0.51
Alcohol use (*n* = 326)	3.29 (7.73)	2.02 (5.70)	−1.28 (7.45)	−3.10 (325)**	−0.17
Cannabis use (*n* = 326)	7.89 (11.83)	6.45 (11.03)	−1.44 (9.79)	−2.65 (325)**	−0.15
Psychological distress (*n* = 326)	0.67 (0.71)	0.47 (0.58)	−0.20 (0.59)	−6.17 (325)***	−0.34
Autonomy (*n* = 324)	4.78 (0.97)	5.05 (0.93)	0.27 (0.99)	4.84 (323)***	0.27
Competence (*n* = 319)	4.69 (1.01)	4.94 (0.99)	0.25 (1.11)	3.97 (318)***	0.22
Relatedness (*n* = 322)	4.97 (0.85)	5.19 (0.77)	0.22 (0.85)	4.70 (321)***	0.26

** *p* < 0.01, *** *p* < 0.001

The number of unmet care needs, arrests by the police, physical health complaints, alcohol use, cannabis use and psychological distress decreased significantly during the same period. Most reported unmet care needs concerned housing related issues (19.7%) followed by finance (13.5%), physical health (11.6%), mental health (8.7%), daily activities (7.7%) and social contacts (4.0%). Most common health complaints were related to the musculoskeletal system and connective tissue (including neck and back complaints) (31.4%), respiratory system (including asthma and chronic obstructive pulmonary disease) (19.8%), digestive system (13.7%) and the circulatory system (including hypertension) (11.3%) and visual (reading, sight) (24.1%), dental (24.1%) and feet (12.5%) problems.

### Predicting perceived health at 18 months after entering the SRS

The univariate relationships between perceived health at 18 months follow-up and the predictor variables are shown in Table 2. Table 3 presents the final model predicting perceived health 18 months after entering the SRS. The percentage of variability in perceived health that could be accounted for by the model was not improved by adding the non-modifiable variables in the third step of the model. The modifiable variables in the final step, however, did add significantly to the predictive power of the model (∆*R* ^2^ = 0.13). More hours of (paid/voluntary) work (*β* = 0.10*)*, a decrease in psychological distress (*β* = −0.19*)* and an increase in competence (*β* = 0.12*)* were significant predictors of perceived health, changes in social support from family and in support from friends and acquaintances were not. The full model successfully explained 38% of the variance in perceived health at 18 months follow-up.

**Table 2:** Univariate relationships between the predictor variables and perceived health at 18 months follow-up, The Netherlands 2011–2013

	1	2	3	4	5	6	7	8	9	10	11
1. Perceived health	1.00										
2. Age in years	−0.15**	1.00									
3. Female	−0.06	−0.22**	10.00								
4. Non-native Dutch	0.02	−0.17**	0.08	10.00							
5. Educational level	0.03	0.24**	−0.11	−0.08	10.00						
6. Suspected intellectual disability	−0.07	0.15**	−0.15**	0.05	−0.06	10.00					
7. Lifetime homelessness	−0.16**	0.10	−0.11	0.09	0.01	0.11	10.00				
8. Company of children	0.06	−0.17**	0.46**	0.12*	−0.07	−0.11*	−0.12*	10.00			
9. Independent housing	0.09	0.02	0.16**	−0.10	0.06	−0.05	−0.25**	0.19**	10.00		
10. Income	0.05	0.08	0.02	−0.02	−0.03	−0.07	−0.03	−0.04	0.03	10.00	
11. Hours of work	0.18**	−0.03	−0.09	−0.03	0.03	−0.08	−0.11*	−0.11*	0.13*	0.10	10.00
12. Social support family	0.15**	−0.06	0.01	−0.02	−0.04	0.01	−0.13*	0.04	0.17**	0.08	−0.02
13. Social support friends	0.18**	−0.07	−0.12*	−0.10	−0.01	0.03	−0.09	−0.02	0.08	0.14*	0.16**
14. Unmet care needs	−0.10	−0.01	0.03	−0.01	0.08	−0.04	−0.05	−0.07	0.06	−0.18**	−0.14*
15. Arrests	−0.01	0.09	0.14*	0.02	0.01	−0.06	−0.08	0.08	0.08	0.09	−0.05
16. Physical health complaints	−0.11	−0.04	−0.08	0.08	0.01	0.01	0.00	−0.06	−0.14**	−0.03	−0.12*
17. Psychological distress	−0.13*	0.07	−0.08	−0.08	0.02	0.01	0.00	−0.09	−0.10	−0.04	−0.18**
18. Alcohol use	−0.10	−0.01	0.05	−0.07	0.04	0.04	−0.08	0.03	0.00	0.08	−0.07
19. Cannabis use	−0.07	0.10	0.13*	0.02	0.05	−0.07	−0.12*	0.14*	0.01	−0.04	0.03
20. Autonomy	0.21**	−0.09	0.03	0.02	0.06	0.06	−0.07	0.09	0.17**	0.07	0.11
21. Competence	0.19**	−0.13*	−0.04	−0.08	0.02	−0.06	−0.06	0.00	0.06	0.20**	0.27**
22. Relatedness	0.08	−0.10	−0.07	−0.01	0.04	−0.07	0.00	−0.05	0.10	0.08	0.05

	12	13	14	15	16	17	18	19	20	21	22
12. Social support family	1.00										
13. Social support friends	0.22**	1.00									
14. Unmet care needs	−0.02	−0.18**	1.00								
15. Arrests	0.03	0.02	0.03	1.00							
16. Physical health complaints	0.00	−0.09	0.17**	0.01	1.00						
17. Psychological distress	0.00	−0.17**	0.32**	−0.01	0.33**	1.00					
18. Alcohol use	0.02	−0.04	0.02	−0.01	0.06	0.14*	1.00				
19. Cannabis use	−0.09	−0.01	−0.09	0.17**	0.08	0.09	0.00	1.00			
20. Autonomy	0.19**	0.26**	−0.25**	−0.08	−0.16**	−0.40**	−0.03	−0.03	1.00		
21. Competence	0.05	0.30**	−0.20**	0.07	−00.10	−0.28**	−0.04	−0.06	0.39**	1.00	
22. Relatedness	0.07	0.32**	−0.14*	0.03	−0.04	−0.30**	−0.13*	−0.03	0.40**	0.34**	1.00

* *p* < 0.05, ** *p* < 0.01

**Table 3:** Summary of a hierarchical regression analysis for variables predicting perceived health at 18 months follow-up (*N* = 327), The Netherlands 2011–2013

	∆*R* ^2^	*B*	SE *B*	*β*	CI
At baseline					
Step 1	0.26***				
Perceived health		0.51	0.04	0.57***	[0.42 to 0.59]
Step 2	0.01				
Age in years		−0.00	0.01	−0.04	[−0.01 to 0.01]
Female		−0.01	0.15	−0.00	[−0.31 to 0.29]
Non-native Dutch		0.02	0.12	0.01	[−0.22 to 0.26]
Upper secondary or higher educational level		0.07	0.14	0.02	[−0.21 to 0.35]
Step 3	0.01				
Suspected intellectual disability		0.01	0.13	0.00	[−0.25 to 0.26]
Duration of homelessness		0.00	0.00	-0.03	[−0.00 to 0.00]
Company of children		0.02	0.25	0.00	[−0.46 to 0.50]
At 18 months follow-up					
Step 4	0.13***				
Changes in hours of (paid/voluntary) work		0.01	0.00	0.10*	[0.00 to 0.15]
Changes in social support family		0.06	0.04	0.06	[−0.03 to 0.14]
Changes in social support friends		0.07	0.05	0.06	[−0.03 to 0.16]
Changes in psychological distress		−0.42	0.12	−0.19**	[−0.64 to −0.20]
Changes in autonomy		0.12	0.07	0.09	[−0.01 to 0.26]
Changes in competence		0.13	0.06	0.11*	[0.01 to 0.25]
Constant		2.60	0.33		[1.96 to 3.24]
Full model	Adj. *R* ^2^ = 0.38				

*CI* confidence interval, *n.a.* not applicable

* *p* < 0.05, ** *p* < 0.01, *** *p* < 0.001

## Discussion

We found a significant improvement in the perceived health of homeless people 18 months after entering the SRS (in the four major cities of The Netherlands). Decreased psychological distress, an increase in the number of hours of (paid/voluntary) work and competence were found to be important predictors of improvement in perceived health. The strongest predictors of perceived health at 18 months were perceived health at entering the SRS and change in psychological distress, while changes in the number of physical health complaints did not account for changes in perceived health. This finding underlines the importance of taking into account the subjective experiences of homeless people in assessing their health.

It is striking that gaining independent housing was not related to changes in perceived health in this study. Previous studies, including Housing First studies, have shown positive health effects when independent housing was combined with support (Gilmer et al. 2010; Hwang and Burns 2014). The effect of housing in this study may have been larger if independently-housed people had been compared with homeless people. Instead, people in independent housing (43.7%) were compared with people of whom a considerable part was housed in residential facilities (29.8%). Furthermore, our sample included a representative sample of all homeless people entering the SRS in 2011 of whom most were homeless for a relatively short period (median 12 months) and did not focus on specific subgroups with serious mental illnesses, substance abuse problems and/or chronic homelessness. This makes comparison with most previous studies difficult (e.g., Gilmer et al. 2010; Hwang and Burns 2014). Finally, differences with the Housing First literature support the notion that Housing First entails more than simply providing housing and emphasizes the importance of providing parallel support, choice and autonomy, minimizing the use of pressure and exploring personal values and goals in addition to actual housing (Tsemberis et al. 2004).

The results of this study did, however, show that a change in the employment situation could predict changes in perceived health. Almost half of the homeless people in this study (44.6%) reported doing some kind of (paid/voluntary) work. In general, participants were satisfied with their work. A lack of work can be understood as an indicator of limited social participation (Vrooman and Hoff 2013), which is negatively related to good mental health (van Straaten et al. 2016b) and physical health (Daiski 2007) among homeless people and the general population in The Netherlands (Jehoel-Gijsbers and Vrooman 2007). Work may also improve health by promoting experiences of self-determination (Ryan and Deci 2000; Ryan et al. 2008). However, it is questionable to what extent the work that is done by these former homeless people provides opportunities to develop a sense of self-determination. In The Netherlands, the poorly-educated are more likely to be unemployed, underemployed or in jobs which are often insecure and lowly-paid. Work is found in the service sectors, (e.g. household and cleaning, sales and health care) (De Graaf-Zijl et al. 2015), areas which provide few opportunities for personal growth.

It is imperative that service providers acknowledge the relative influence of potential modifiable factors on the perceived health of homeless people. To do so would enable them to prioritize the types of service provision offered, while taking into account the limited financial resources and extensive care needs of the homeless population. Our findings suggest that social services should aim to increase the experiences of competence of those entering the SRS. An autonomy-supportive care climate stimulates autonomy and competence and in turn facilitates better mental and physical health (Ryan et al. 2008). Eliciting the clients’ perspective, providing choice and information, minimizing the use of pressure to change and promoting competence are important factors for autonomy support (Williams et al. 2002). Furthermore, competence can be supported by motivational interviewing (Markland et al. 2005). Meaningful activities, such as training programmes or volunteering are an important means of developing new skills and promoting a feeling of competence, especially when finding paid employment may not be realistic (Patterson et al. 2015). Meanwhile, society should create the conditions in which vulnerable people are provided with opportunities to build on their potential (Wolf 2016). It is important for service providers to realize that individual programme plans for homeless people are not only a means of attaining stable housing, income, work and contact with service providers. They should also provide an opportunity to explore the personal values and goals of the client, as this in itself can promote an increased satisfaction with life (Shern et al. 2000). Such goals should be appropriately challenging to one’s people’s skills and experience levels.

The strengths of our study include its longitudinal design and the inclusion of a client perspective, which has often been lacking in studies on homelessness. A first limitation was the selective non-response at 18 months follow-up that included younger participants and participants with the lowest level of education at baseline. Because age was negatively related to perceived health in the univariate analysis, selective non-response to follow-up may have biased our findings by resulting in an underestimation of the perceived health of our participants. Second, to explore the relationship between changes in the modifiable variables and changes in perceived health we created independent variables that represented the change or improvement in each client by subtracting 18 months follow-up measures from the baseline measure of that variable. Participants with high scores at both interviews were not differentiated from those with low scores at both time points. Table 1 shows low mean baseline scores and significant improvements in all variables, leading us to believe this was the best strategy for this particular research question. Third, this study has missing values on variables and scales (4.6% at the highest for changes in social support) because participants could choose to refrain from answering questions. Therefore, we used mean substitution in the hierarchical regression analysis to prevent a possible bias which might have occurred if we had only selected those participants who chose to answer all the questions. This is the reason why the correlations involving the variable(s) that are imputed are attenuated, giving a conservative estimation of the relations and providing a rigorous test of the relationships. To explore the effect of this conservative estimation we also ran the analysis without mean substitution and found similar results (available upon request), except for a significant relationship between autonomy and perceived health (*β* = 0.11, *p* = 0.044). Fourth, the reliability of the competence scale was relatively low. Homeless people experience a strong sense of learned helplessness when they lose the belief that their own actions can influence the course of their lives (Wolf 2016). This could lead to a less consistent pattern of reactions to the competence questions in comparison with the general population (Johnston and Finney 2010) on items including: “Often, I do not feel very competent”. and “In my life I do not get much of a chance to show how capable I am”. Therefore, these first interesting findings on improvements in competence and the positive relationship between perceived health and competence in this population needs further exploration before translating it into practice for social policy or services provision. Finally, substantial subgroups of the sample of this study had a suspected ID, a foreign background and/or a lower educational level. This raises questions about the reliability of perceived health for these subgroups. However, additional analyses (available upon request) showed sufficient reliability (Cronbach’s *α* ≥.70) for all the subgroups.

### Conclusions

This study clearly demonstrates that perceived health is not only influenced by objective circumstances related to work and mental health, but also by self-determination, as shown by the influence of competence. Services should, therefore, aim to reduce psychological distress, to support homeless people in increasing their working hours, with voluntary work if paid employment is not available. They should also focus on the strengthening of the competence of homeless people to improve their perceived health. A valuable tool to help attain these goals could be individual action plans that take into account the client’s personal values and experiences, thereby strengthening their competence.
